# Extensive genomic rearrangements mediated by repetitive sequences in plastomes of *Medicago* and its relatives

**DOI:** 10.1186/s12870-021-03202-3

**Published:** 2021-09-14

**Authors:** Shuang Wu, Jinyuan Chen, Ying Li, Ai Liu, Ao Li, Mou Yin, Nawal Shrestha, Jianquan Liu, Guangpeng Ren

**Affiliations:** 1 State Key Laboratory of Grassland Agro-Ecosystems, Institute of Innovation Ecology & School of Life Sciences, Lanzhou University, Lanzhou, China; 2 Key Laboratory of Bio-Resource and Eco-Environment of Ministry of Education &State Key Lab of Hydraulics & Mountain River Engineering, College of Life Sciences, Sichuan University, Chengdu, China

**Keywords:** *Medicago*, *Trigonella*, *Melilotus*, IRLC, Plastome evolution, Genomic rearrangement, Repeat

## Abstract

**Background:**

Although plastomes are highly conserved with respect to gene content and order in most photosynthetic angiosperms, extensive genomic rearrangements have been reported in Fabaceae, particularly within the inverted repeat lacking clade (IRLC) of Papilionoideae. Two hypotheses, i.e.*,* the absence of the IR and the increased repeat content, have been proposed to affect the stability of plastomes. However, this is still unclear for the IRLC species. Here, we aimed to investigate the relationships between repeat content and the degree of genomic rearrangements in plastomes of *Medicago* and its relatives *Trigonella* and *Melilotus*, which are nested firmly within the IRLC.

**Results:**

We detected abundant repetitive elements and extensive genomic rearrangements in the 75 newly assembled plastomes of 20 species, including gene loss, intron loss and gain, pseudogenization, tRNA duplication, inversion, and a second independent IR gain (IR ~ 15 kb in *Melilotus dentata*) in addition to the previous first reported cases in *Medicago minima*. We also conducted comparative genomic analysis to evaluate plastome evolution. Our results indicated that the overall repeat content is positively correlated with the degree of genomic rearrangements. Some of the genomic rearrangements were found to be directly linked with repetitive sequences. Tandem repeated sequences have been detected in the three genes with accelerated substitution rates (i.e.*, accD, clpP*, and *ycf1*) and their length variation could be explained by the insertions of tandem repeats. The repeat contents of the three localized hypermutation regions around these three genes with accelerated substitution rates are also significantly higher than that of the remaining plastome sequences.

**Conclusions:**

Our results suggest that IR reemergence in the IRLC species does not ensure their plastome stability. Instead, repeat-mediated illegitimate recombination is the major mechanism leading to genome instability, a pattern in agreement with recent findings in other angiosperm lineages. The plastome data generated herein provide valuable genomic resources for further investigating the plastome evolution in legumes.

**Supplementary Information:**

The online version contains supplementary material available at 10.1186/s12870-021-03202-3.

## Background

In photosynthetic angiosperms, plastid genomes (plastomes) are highly conserved in gene content and structure and exhibit quadripartite structure with a pair of inverted repeats (IRs), which separate large single copy (LSC) and small single copy (SSC) regions [1, 2]. The plastomes of angiosperms typically consist of approximately 80 protein-coding genes, which play roles in photosynthesis and housekeeping along with 30 tRNA and 4 rRNA genes [3], of which approximately 17 genes are duplicated in the IR region. Because of the advent of high-throughput sequencing technologies, over 4500 land plant plastomes have been sequenced since the first tobacco plastome published in 1986 [4] and are publicly available in NCBI (accessed November 19, 2020). The size of these plastomes ranges from 16 to 242 kb. The majority of land plant plastomes range from approximately 110 to 170 kb, and the variation in plastome size is often attributed to IR expansion, contraction, or loss [5, 6]. The most enormous IR expansion is found in *Pelargonium transvaalense* (Geraniaceae) [7], where the IR expanded more than three times (87.7 kb) compared with the usual size of IR (~ 25 kb). On the opposite extreme, the IR loss, which causes reduction in plastome size, have been documented in many independent lineages, including two lineages of *Erodium* (Geraniaceae) [8, 9], *Carnegiea gigantean* (Cactaceae) [10], *Tahina spectabilis* (Arecaceae) [11], the Putranjivoid clade of Malpighiales [12] and the IR-lacking clade (IRLC) of Papilionoideae (Fabaceae) [13].

Although IR loss seems to be more common than previously thought, the presence of IR across angiosperms is still predominant, suggesting its functional importance in angiosperms. Early findings, which suggest that lineages lacking the IR have undergone more frequent genomic rearrangement than those that have retained the IR, support the hypothesis that IR plays a role in stabilizing plastome structure [13–15]. However, recent studies on the plastomes of Oleaceae [16], *Erodium* [8], *Pelargonium* [5–7], and *Plantago* [17] showed that the presence of IR does not ensure genome stability. Instead, the genome stability is more correlated with the overall repeat content in *Erodium* [8]. Other functions of IR, such as conservation of genes encoding the translational machinery [13] have also been suggested, with regard to the fact that substitution rates of genes in the IR are approximately three-fold slower than those in the single-copy (SC) regions [17–19]. However, this pattern of reduced IR substitution rates does not apply universally to many other plants. The IR genes from species in the genera *Pelargonium*, *Plantago,* and *Silene* have different levels of add substitution rates compared with the SC genes, which result from a mixture of locus-specific, lineage-specific, and IR-dependent effects [7, 17]. Increased locus-specific rates have been observed in plastomes of many plants [13, 14, 17, 20, 21], and such mutation hotspots are suggested to be linked to increased recombinational activities, which are likely driven by the proliferation of repeats. Consequently, repetitive DNA may have played an important role in structural variations of plastomes.

Previous studies have suggested that some species of the IRLC have acquired dramatic variations in plastome structures, including abundant inversions, mutation hotspots, gene transfers of *rpl22, infA*, substitution of *rps16*, losses or pseudogenization of *accD* and *ycf4*, IR reemergence, and losses of two *clpP* introns during their evolution [11, 20, 22–24]. Many of these rearrangement events have been also reported in *Passiflora* plastomes [25–28]. Illegitimate recombination between homologous and/or homeologous sequences within and between unit genome copies is proposed to yield structural variations in plastomes [29, 30]. Despite the IR losses, there are no clear signals of illegitimate recombination found in the IRLC species, or at least none remains as the IR loss is not recent [8]. A direct link between recombination and mutation hotspots was reported from the IR-lacking plastome of *Lathyrus*, in which *c.* 1.5 kb localized hypermutation region around *ycf4* was caused by repeated DNA breakage and repair [20]. In addition, repeat-mediated recombination-dependent replication has caused a ~ 9 kb IR reemergence in *Medicago minima* [11, 31]. Notably, plastomes of the IRLC species have abundant repetitive DNA and this seems to be rare in angiosperms’ chloroplast DNA (cpDNA) [22]. However, whether the content of repetitive DNA is correlated with structural variations, and if yes, how repetitive DNA affect plastomes variations in the IRLC species remains understudied.

In this study, we focus on *Medicago* (*M*.) L. and its relatives *Trigonella* (*T*.) L. and *Melilotus* Miller, all belonging to the tribe Trifolieae, which is nested firmly within the IRLC. Species of these three genera are very important legume forage with significant ecological and economic values, including the widely cultivated major forage crop species *M. sativa*, the legume model species *M. truncatula* and the widely cultivated medicinal species *T. foenum-graecum*. Choi et al. [11] completed the plastomes of 19 *Medicago* species and one *Trigonella* species and revealed modest structural variations among them, but their discussion focused mainly on the IR reemergence in *M. minima*. Here, we took advantage of whole-genome resequencing data and assembled the plastomes of 75 individuals representing 20 species in the three genera. We aimed to characterize plastome structural variations of the 20 species at multiple individual levels and investigate the correlations between structural variations and repetitive elements.

## Results

### Plastome features

The plastome sequences were assembled and annotated for 75 individuals representing 20 species. Sizes of the plastomes ranged from 121,043 bp (*M. orthoceras*) to 142,713 bp (*Melilotus dentata*), and numbers of unique annotated genes from 110 to 111 (see Table S1). The total genes included 75–76 unique protein-coding genes (PCGs), 30 unique transfer RNA (tRNA) genes and 4 unique ribosomal RNA (rRNA) genes (Fig. S1). We found that the GC content of all plastomes ranged from 33.6 to 34.1% (Table 1; Table S1).

**Table 1:** Information of plastome assembly, annotation, number of genomic rearrangements, and percent repetitive elements

Genus	Species	No. of individuals	Entire	Overall		No. of	No. of rRNA genes	No. of tRNA genes	No. of genomic rearrangements	Percent repetitive DNA (%)^**b**^
			plastome	GC	No. of	Protein-				
			size	content	genes	coding				
			(bp)	(%)		genes				
***Medicago***	*M. polymorpha*	5	124,247-124,445	34.0, 34.1	110	75	4	30	5	4.41–4.56
	*M. truncatula*	2	123,391-123,767	34.0	110	76	4	30	4, 5	4.00–4.16
	*M. sativa*	3	125,523-125,623	33.8, 33.9	110	76	4	30	4	4.13–4.55
	*M. lupulina*^*a*^	3	122,194-122,310	34.1	110	76	4	30	9	5.93–6.02
	*M. minima*^*a*^	5	132,071-132,219	34.2	110	75, 76	4	30	7, 8	4.99–5.06
	*M. ruthenica*	5	127,065-127,674	34.2	112	76	4	32	8	5.53–6.07
	*M. archiducis-nicolai*	4	126,635-126,810	34.1	112	76	4	32	7	5.20–5.38
	*M. platycarpos*	2	125,502-125,528	34.1	110	76	4	30	6	4.27
	*M. falcata*	3	125,357-125,555	33.8, 33.9	110	76	4	30	5	4.17–4.22
	*M. edgeworthii*	5	122,454-122,549	33.9, 34.0	110	76	4	30	4	4.01–4.09
	*M. monantha*	3	121,336-121,358	34.1	110	76	4	30	5	3.43–3.46
	*M. orthoceras*	5	121,043-121,065	34.1	110	76	4	30	5	2.98–3.11
	*M. arcuata*	2	121,728-121,777	34.0	110	76	4	30	5	3.54–3.57
	*M. cancellata*	5	121,889-121,953	34.0	110	76	4	30	5	3.57–3.63
***Trigonella***	*T. cachemiriana*	3	125,555	34.0	110	76	4	30	6	3.96–4.04
	*T. emodi*	5	128,493-128,643	33.8	110	76	4	30	9	6.19–6.29
***Melilotus***	*Melilotus dentata*^*a*^	4	141,922-142,713	33.7, 33.8	111	76	4	30	6	4.62–4.94
	*Melilotus indicus*	3	127,703-128,044	33.6	112	76	4	31	7	6.07–6.39
	*Melilotus officinalis*	4	126,534-127,451	33.7	111,112	76	4	31	5, 6	5.43–6.13
	*Melilotus alba*	4	127,293-127,694	33.6, 33.7	111, 112	76	4	31	6, 7	5.96–6.25
**Total**	**20**	**75**	**121,043-132,219**	**33.6–34.1**	**110–112**	**75–76**	**4**	**30–32**	**4–9**	**2.98–6.39**

^a^ GC content, No. of genes, protein-coding genes, rRNA genes, tRNA genes, and Percent repetitive DNA were calculated using only one IR copy

^b^Tandem repeats and dispersed repeats ≥30 bp

### IR reemergence in *Melilotus dentata*

**Fig. 1:**
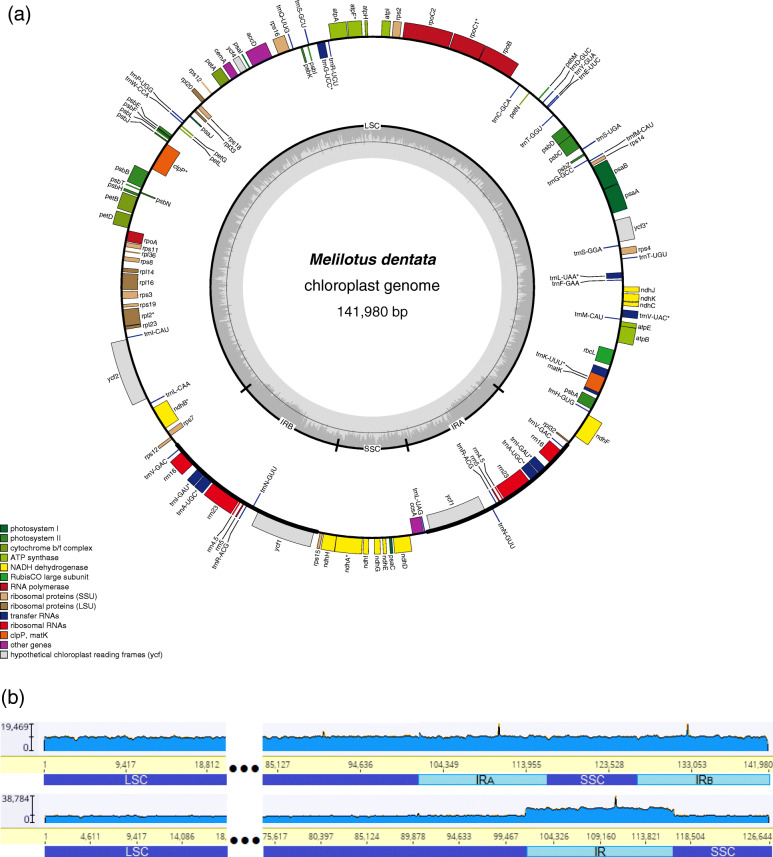
Circular representation of *Melitotus dentata* plastome and confirmation of IR extent in it. **a** Circular representation of *Melitotus dentata* plastome. Genes shown outside the circle are transcribed clockwise and those inside are transcribed counter clockwise. Genes belonging to different functional groups are color-coded. The dark gray area in the inner circle indicates GC content and the thick black line shows the extent of different regions. LSC: large single copy; SSC: small single copy; IR: inverted repeat; IRA: inverted repeat A; IRB: inverted repeat B. **b** Confirmation of IR extent in *Melilotus dentata*. Whole genome resequencing reads were mapped to the assembled *Melilotus dentata* plastome containing both two copies (upper) or a single copy (lower) of the novel IR. The scale at the left reports the depth of reads, which is indicated graphically by the blue histogram

Assembly and annotation of plastomes of *Melilotus dentata* using a series of parameters (see Materials and methods) suggested the presence of a large inverted repeat (~ 15 kb, ranging in size from 15,336 bp to 15,553 bp), which contained 10 coding genes, including *ycf1*, the conserved four rRNA genes (4.5S, 5S, 16S, and 23S rRNA) and five tRNA genes (*trnR-*ACG*, trnN-*GUU*, trnA-*UGC*, trnI-*GAU, and *trnV-*GAC) in the seed plants (Fig. 1a; Fig. S2). We preformed read mapping to confirm the novel ~ 15 kb IR assembled in *Melilotus dentata*. The whole genome resequencing reads of *Melilotus dentata* were mapped to the assembled plastome sequence. Visualization of the mapping result (Fig. 1b) showed even distribution of reads over the assembled plastome sequence when both copies of the IR are included and ~ two-fold higher depth of coverage over the IR region compared with SC regions when only one IR copy is considered. This adds another evidence to the IR reemergence in the IRLC in addition to previous findings in *M. minima* [11, 31]. The IR reemergence in *Melilotus dentata* was further confirmed by multiple individuals, but with some INDELs (insertions and deletions) between the two IR copies in *Melilotus dentata* 02 and *Melilotus dentata* 03 (Fig. S2). We found that the size variation of the two IR copies was due to the difference in the copy number of tandem repeats (Fig. S2).

### Plastome structural variations

**Fig. 2:**
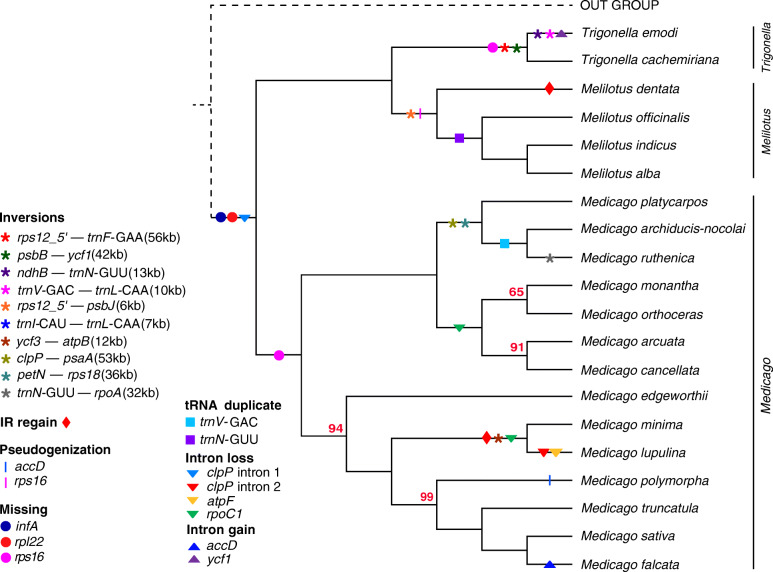
Phylogenetic relationships and distributions of plastome structural variations. Structural features of all 20 taxa are plotted on the branches of the cladogram inferred using PhyML and indicated in the key (inset) and inversions are relative to *Wisteria floribunda*. The red number at the node indicated bootstrap values < 100. The branch of the outgroup was indicated by dotted line and did not participate in the statistics and labeling of structural variation. Structural features for all individuals are listed in Table S1

The plastome structural variations, including three IR regain*,* two pseudogenization, three gene loss, four intron loss, two intron gain, two tRNA duplicate, and 10 inversions (using *Wisteria floribunda* as the reference, see Fig. S3), were detected among the 20 species (Fig. 2; Table S1). Structural variations among individuals within each species were mostly the same, except for the pseudogenization of *ycf2* (presence of premature stop codons within the gene) in *M. minima* 01, duplicate of *trnN*-GUU in some individuals of *Melilotus officinalis* and *Melilotus alba* (Table S1), and two distinct plastome configurations in *M. truncatula* (Fig. S1k; Table S1). Most of the variations were shared by two to multiple species, while some of them were specific to certain species. The *rpl22* and *infA* were absent in all the 20 species, whereas *rps16* was lost in the *Medicago* and *Trigonella* clades, and pseudogenized (presence of premature stop codons within the gene) in the *Melilotus* clade*.* Two kinds of duplicated tRNA were present in three *Melilotus* and two *Medicago* species. The tRNA duplicates were confirmed by read mapping as shown in Fig. S4. The intron loss of *clpP*, *atpF* and *rpoC1* in *M. lupulina* and *M. minima* was consistent with previous findings [11]. The intron 1 of *clpP* was lost in all the 20 species, consistent with previous studies [23, 32]. We further found that the intron loss of *rpoC1* was shared by six *Medicago* species (Fig. 2; Table S1). After validation based on transcriptomic data (see more details in Methods), the intron gain of *ycf1* and *accD* were specific to *T. emodi* and *M. falcata*, respectively. Only one gene pseudogenization (*accD*, truncated sequence) was unique to *M. polymorpha*, and others were shared by multiple species, mostly by closely related species. For inversions, the large ones (> 30 kb) occurred mainly in two groups: the *Trigonella* clade and the clade containing *M. platycarpos*, *M. ruthenica*, and *M. archiducis-nocolai* (section *Platycarpae*). For the remaining inversions, two were unique to *T. emodi*, two were present in the *Melilotus* clade, and one was shared by *M. lupulina* and *M. minima*.

### Repeat analysis

The analysis of the 75 plastomes recognized 64–597 pairs of dispersed repeats (including forward, reverse, palindromic and complement repeat), ranging in size from 2550 to 7315 bp (Table S2). The most abundant repeat type was forward repeats with the number ranging from 40 pairs in *M. orthoceras* to 571 pairs in *Melilotus indicus*. The second abundant repeat type was palindromic repeats ranging from 17 pairs in *M. edgeworthii, M. truncatula* 02, and *T. cachemiriana* to 47 pairs in *M. truncatula* 01. Then a pair of reverse repeats in *M. polymorpha*, *M. edgeworthii*, *M. monantha*, and *Melilotus dentata* to 50 pairs in *Melilotus officinalis*. Lastly, the complimentary repeats were infrequent among the species ranging from 1 to 7 while others did not have such as *M. polymorpha*, *M. truncatula*, *M. sativa*, *M. archiducis-nicolai*, and *M. falcata*. A total of 38–110 tandem repeats were recognized across the 75 plastomes with their sizes ranging from 2033 to 5951 bp (Table S2).

**Fig. 3:**
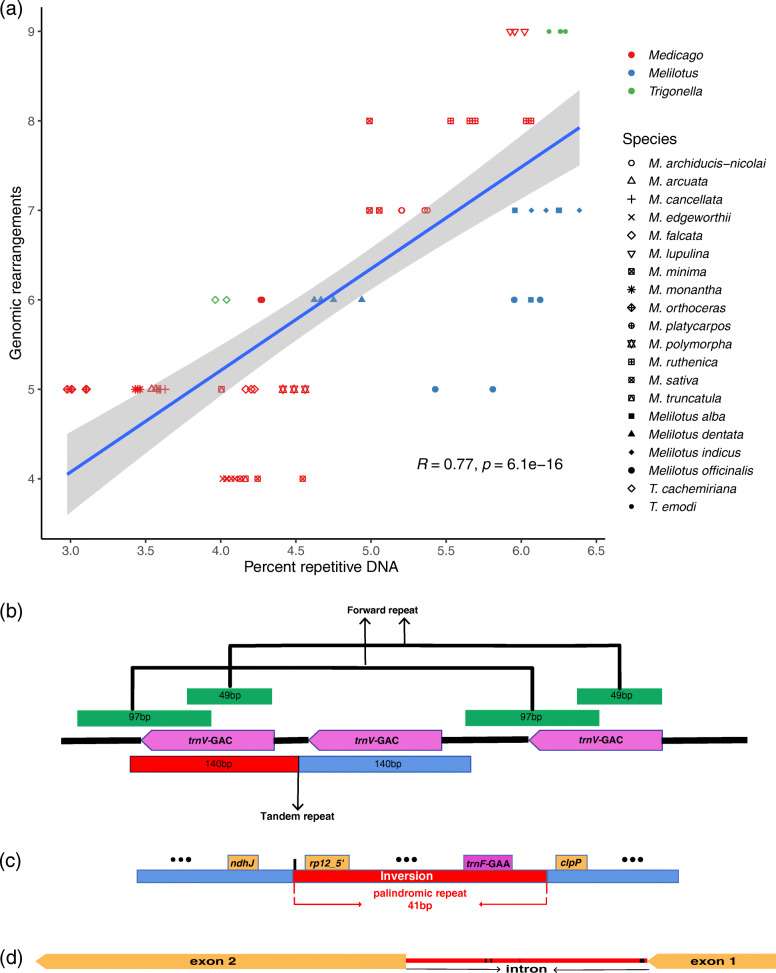
Relationships between repetitive sequences and plastome structural variations. **a** The overall repeat content shows significant positive correlation with the degree of genomic rearrangements (*R* = 0.77, *P* < 6.1e-16). **b**-**d** Examples of repeat-mediated tRNA duplicate, inversion and intron gain. The gained intron is constituted mostly by repeats (red)

Overall, we identified 3604 (*M. orthoceras*) to 8179 bp (*Melilotus indicus*) repetitive sequences across the 75 plastomes, accounting from 2.98 to 6.39% of their full plastomes, respectively (Table 1, Table S2). We found that the overall repeat content showed significant positive correlation with the degree of genomic rearrangement (*R* = 0.77, *P* < 6.1e-16; Fig. 3a). Among the repeats, both the dispersed repeats and tandem repeats also showed significant positive correlation with these structural variations (Fig. S5). Some of the genomic rearrangements were found to be directly linked to repetitive sequences (Fig. 3b-d; Figs. S6-S8; Tables S3-S6). For example, the three copies of *trnV-*GAC in *M. archiducis-nicolai* were linked to two forward repeats and one tandem repeat (Fig. 3b; Fig. S6a; Table S3). The detected 56 kb inversion (*rps12_5’—trnF-*GAA) in *T. cachemiriana* had a pair of 41 bp inverted repeats flanked to its two endpoints (Fig. 3c; Fig. S7a; Table S4; Table S5). Such short inverted repeats were also present in the 32 kb inversion (*trnN-*GUU*—rpoA*) in *M. ruthenica* and the 36 kb inversion (*petN—rps18*) in *M. archiducis-nicolai, M. ruthenica* and *M. platycarpos* (Fig. S7i, j; Table S4; Table S5). Furthermore, *M. truncatula* 02 had a ~ 44-kb inversion compared to the *M. truncatula* 01, mediated by a short, imperfect repeat (Fig. S1k; Table S1), which is consistent with the findings of Gurdon and Maliga [33]. In addition, the gained intron mainly consisted of repetitive sequences (Fig. 3d; Fig. S8; Table S6).

### Comparative plastome analysis

**Fig. 4:**
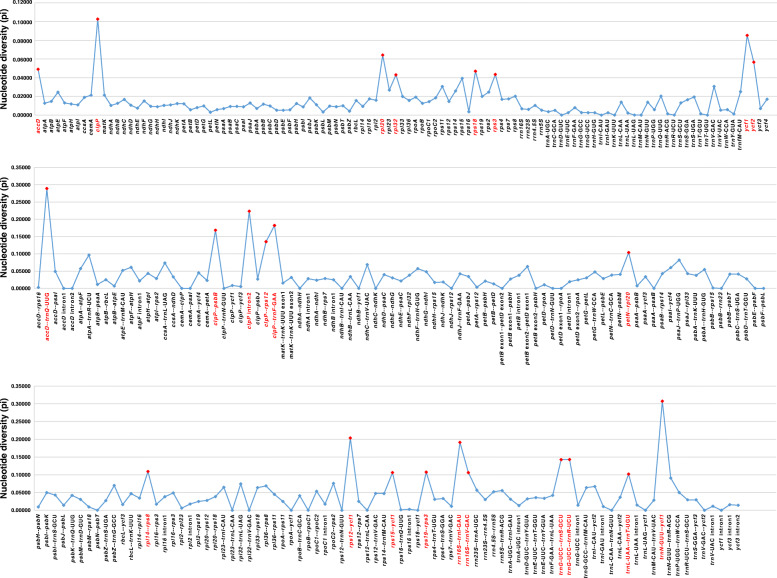
Comparison of nucleotide diversity (Pi) among the 20 species. X-axis: names of genes or intergenic regions, Y-axis: nucleotide diversity (Pi) of each region. We identified 8 genes (Pi > 0.04) and 16 intergenic regions (Pi > 0.1) as highly divergent regions and colored them with red

To display interspecific variations graphically, the sequence variations of the 20 species was generated using mVISTA with plastome of *M. falcata* as the reference (Fig. S9). The most highly divergence regions among the 20 species appeared mostly in the intergenic spacers, while the coding regions showed relative conservatism except for the genes *clpP, atpF*, *rpoC1*, *accD, ycf1* and *ycf2*. As a result, we identified eight highly divergent coding regions (π > 0.04) and 16 highly divergent non-coding regions (π > 0.1) (Fig. 4; Table S7).

### Acceleration of substitution rates in *accD*, *clpP,* and *ycf1*

**Fig. 5:**
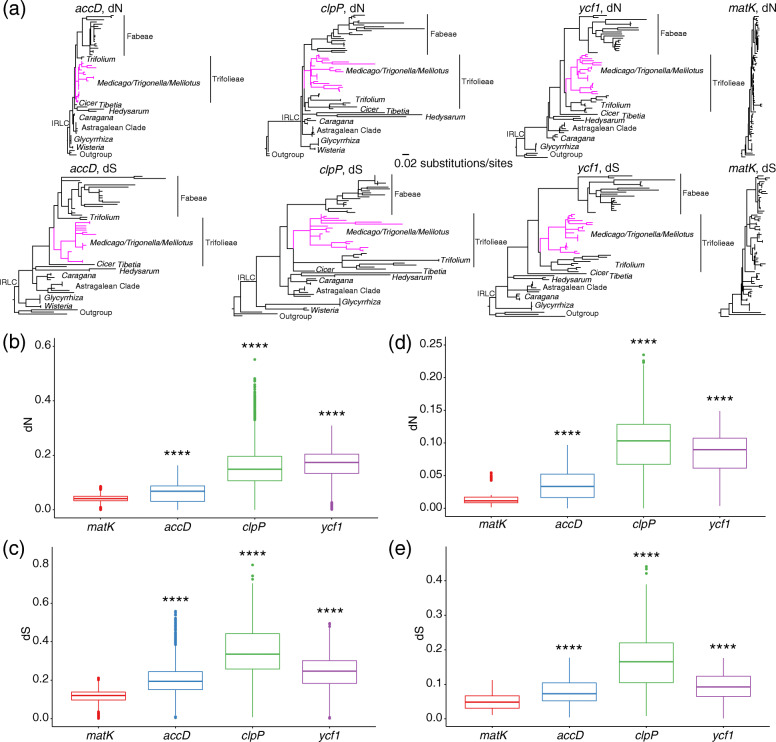
Acceleration of substitution rates in *accD*, *clpP* and *ycf1.* **a** Synonymous (dS) and nonsynonymous (dN) divergence in the IRLC species for four chloroplast genes: *accD*, *clpP, ycf1* and *matK*. Shown are dN (upper) and dS (lower) trees resulting from a codon-based maximum likelihood (ML) analyses performed by RAxML, rooted using three Robinioid sequences (non-IRLC species): *Lotus japonicus, Sesbania grandiflora* and *Robinia pseudoacacia*. All trees are drawn to the same scale. The species are in the same order from top to bottom in dN and dS trees of each gene, to the greatest extent possible, and are named in full in Fig. S11. Pink branches in the trees for *accD*, *clpP,* and *ycf1* represent species in our study. Trees for *matK* do not show comparable rate heterogeneity at either synonymous or nonsynonymous sites. **b**-**e** Significance test for nonsynonymous (dN) (upper) and synonymous (dS) (lower) substitution rates of *clpP*, *accD* and *ycf1* compared to *matK* for all the comparisons within both IRLC taxa used in this study (left) and our own 20 species (right). ****, *P* < 0.0001 (T-test). Detailed information can be found in Tables S8-S11

**Fig. 6:**
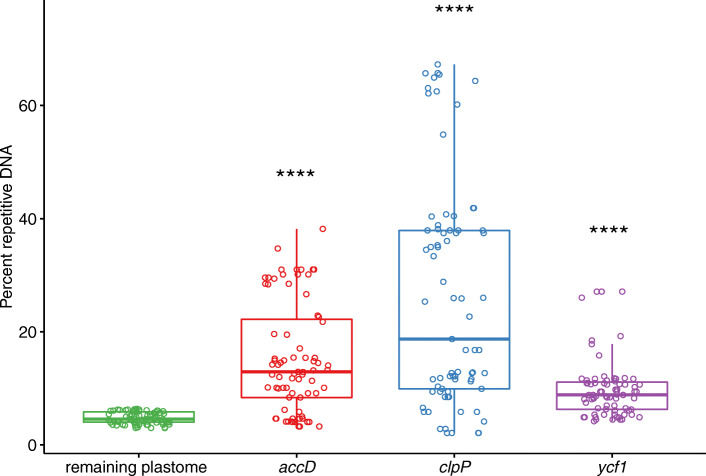
Repeat content of the three localized hypermutation regions around the three genes (*accD, clpP,* and *ycf1*) compared to the remaining part of plastome sequences across the 75 individuals. ****, *P* < 0.0001 (T-test). Detailed information can be found in Table S14

We found that there were significant increases (*P* < 0.0001) in the substitution rates (dN and dS) of *clpP*, *accD*, and *ycf1* compared to *matK* and *rbcL* for all the comparisons within both IRLC taxa and our own 20 species (pink branch) (Tables S8-S12; Fig. 5; Fig. S10). Furthermore, all of the investigated IRLC species including our 20 species showed signs of various degrees of elevated branch lengths in the dN and dS trees of *accD*, *clpP*, and *ycf1*, but again no similar pattern was seen in *matK* and *rbcL* (Fig. 5; Fig. S11). The three genes also exhibited high variations in terms of their coding sequence (CDS) length compared with *matK* and *rbcL* in all the 20 species (Table S13). The CDS length of *accD* varied from 1299 bp (*M. falcata*) to 2190 bp (*M. minima*); *clpP* from 582 bp (*M. falcata*) to 684 bp (*M. ruthenica*) and *ycf1* from 4893 bp (*Melilotus alba* and *Melilotus indicus*) to 5352 bp (*M. lupulina*), while the length of the other two genes did not show much variation (1512 or 1521 bp in *matK* and 1428 bp in *rbcL*) (Table S13). To investigate the mechanisms of length-associated mutation for CDS of these three genes, we counted the number and overall length of repetitive elements in the CDS of all the five genes (Table S13). As expected, *accD* and *ycf1* in 20 species all included tandem repeated sequences whose numbers were positively correlated with gene length. The longest length of *clpP* was found in *M. ruthenica*, which also included a tandem repeated sequence. However, we did not detect any tandem repeated sequence in *matK* and *rbcL*. We also found that the repeat contents within these genes and their neighboring hypermutable regions (π > 0.1) (Fig. 4) were significantly higher (*P* < 0.0001) than that of the remaining plastome sequences (Fig. 6; Table S14). Therefore, the acceleration of substitution rates of these three genes may be explained by the insertions of repetitive sequences.

## Discussion

### IR reemergence in *Melilotus dentata*

The existence of a pair of inverted repeats is a feature of plastomes throughout the 400 million years of land plant evolution [34] with the exception that a copy of IR is absent in some lineages, such as the IRLC [35], some species of *Erodium* (Geraniaceae) [8, 14], Pinaceae [36], and *Carnegiea gigantea* [10]. In addition to the first ~ 9 kb IR reemergence (containing 7 coding sequences) found in *M. minima* in the IRLC [11], we reported here the second case in *Melilotus dentata*, in which ~ 15 kb of a large inverted repeat is regained, containing 10 coding sequences compared to the ~ 17 in the typical IR of angiosperms. Choi et al. [11] speculated that the IR reemergence in *M. minima* occurs via synthesis-dependent strand annealing or the formation and resolution of Holiday junctions during recombination-dependent DNA repair. Both processes need to be mediated by repeats. Choi et al. [11] detected unique variations (i.e., two pairs of inverted repeats flanked by *rps12_5’* and between *trnN-*GUU and *ycf1*, and a series of tandem repeats between *trnN*-GUU and *ycf1*) retained in *Medicago suffruticosa,* which is closely related to *M. lupulina* and *M. minima*. The position of repeats in *M. suffruticosa* flanked the endpoints corresponding to the novel IRs in *M. minima* suggests that their ancestor may have experienced repeat-mediated perturbations, which may have caused IR regain in the two species. Repeat-mediated and recombination-dependent replication play a significant role during the IR reemergence [11]. However, we did not detect a similar pattern in *Melilotus dentata* and its relatives, which may be due to incomplete sampling in our study. Dynamic characteristics of IR boundary migration process [17, 37] and the IR regain in *M. minima* and *Melilotus dentata* supports the hypothesis that the novel IRs are quite possible to expand continuously and include more adjacent canonical IR genes gradually [11]. A deeper and wider sampling of the IRLC species is necessary to further investigate how many species of the IRLC have regained novel IR and the mechanism behind this process.

### Acceleration of substitution rates in *accD*, *clpP,* and *ycf1* are related to repetitive sequences

Gene-specific rate acceleration has been frequently reported in plastome evolution. Among legumes, *clpP* and *ycf1* have been reported as rapidly evolving genes in Papilionoids [38]. The gene *clpP* is also accelerated in Mimosoid [24] and *ycf4* is accelerated in most legumes especially in the tribe Fabeae [20, 39]. Accelerated evolutionary rate of *accD* in *Jasminum* [16], *Silene* [38], and *Plantago* [17] has also been described. In agreement with previous findings, our results indicate that the three genes exhibit different degrees of substitution rate acceleration (Fig. 5; Figs. S10, S11) with high variations in point mutations and length as compared to *matK* and *rbcL* (Fig. 4; Tables S7-S13). The gene *accD* encodes acetyl-CoA carboxylase, which acts in fatty acid biosynthesis [40] and *clpP* encodes a protein, which is a part of multimeric protease [41]. For *ycf1,* recent experiments suggest that it encodes Tic214, which is a vital component of *Arabidopsis* translocon on the inner chloroplast membrane (TIC) complex, thus it is essential for plant viability [42]. Notably, tandem repeated sequences have been detected in these three genes and their length variation could be explained by the insertions of tandem repeats (Table S13). Interestingly, our results suggest that the overall repeat contents within the three genes with accelerated substitution rates and their neighboring hypermutable regions (π > 0.1) were significantly higher than that of the remaining plastome sequences (Fig. 6; Table S14). It is possible that the repetitive elements inserted into or near the three genes might have promoted them to become more variable, resulting in the acceleration of the substitution rate.

### Plastome structural variations are mediated by repetitive sequences

Although the gene content and order of plastome is highly conserved in most seed plants [1], extensive genomic rearrangements have mostly been reported in Fabaceae [22, 23, 43, 44], particularly within the IRLC [23]. Indeed, we detected abundant plastome structural variations, including three IR regains, three gene losses, two pseudogenization, four intron losses, two intron gains, two tRNA duplications, and ten inversions in the 20 species (Fig. 2; Table S1). Among these variations, some are reported in previous studies too. For example, the loss of *rpl22* and *infA* found in the 20 species and also in other legumes [45] and almost all rosids [46] have been suggested as successful gene transfers from the plastome to the nuclear genome. Similarly, the loss or pseudogenization of *rps16* in the 20 species could be explained by gene substitution as suggested for *M. truncatula* [47].

With these extensive genomic rearrangements, we must consider what factors might contribute to the plastome instability in these species. The hypothesis that IR plays an important role in stabilizing plastome structure may be one possible explanation because all our 20 species are defined as IR-lacking species within the IRLC [13–15]. However, relatively higher structural variations were detected in the two IR reemergence species (*M. minima* and *Melilotus dentata*, IR > 9 kb) compared with other species (Fig. 2), such as *M. truncatula, M. sativa*, and *M. edgeworthii*, do not support this hypothesis. With more available plastome sequences, the recent findings in a few unrelated lineages suggested that neither loss of the IR has destabilized the plastome nor the presence of the IR has ensured genome stability [7, 8, 16, 17]. Instead, the accumulation of repeat sequences may be more important to plastome stability as suggested by many recent studies [8, 20, 48].

We did find abundant repetitive elements in the 20 species and a significant positive correlation between the overall repeat content and the degree of genomic rearrangements (Fig. 3a). Moreover, the dispersed repeats likely contributed more to these variations than the tandem repeats (*R* = 0.73 vs. 0.48; Fig. S5). Specifically, we found a high frequency of repeats flanked the endpoints of most inversions (Fig. 3c; Fig. S7; Tables S4, S5), and the gained introns were constituted mainly by repeats (Fig. 3d; Fig. S8; Table S6). Furthermore, the two tRNA duplicates were mediated by tandem repeats and/or forward repeats (Fig. 3b; Fig. S6; Table S3). Many previous studies have also reported that repetitive sequences are commonly found in the flanking regions of inversions, losses, and tRNA duplicates in the plastomes of angiosperms [14, 33, 48–50]. Repeat-mediated illegitimate recombination is thought to be one of the major mechanisms leading to these genomic rearrangements [51, 52]. As recombinogenic substrates, when a plastome’s repeat content increases, differential resolution of recombination events (including illegitimate recombination) between highly similar regions (e.g., repeats) within and between unit genome copies become more likely, which may ultimately result in a rearranged plastome.

Although it is unclear how repetitive elements are generated, our results suggest that their appearances and losses are a dynamic process and are random on an evolutionary timescale. Given the fact that most of the genomic rearrangements are species-specific or only shared by closely related species, if all these different variations are due to repeat-mediated illegitimate recombination, then those repeats must be different and their appearances should be random during the evolutionary histories of these species. Similarly, repeats may be lost randomly. One contrary assumption is that if losses of repeats are progressively on a timescale, then we would expect a similar repeat pattern flanked the endpoints of genomic rearrangements on the same evolutionary scale. However, the two inversions (Fig. S7a, b) shared by *T. cachemiriana* and *T. emodi*, and another two inversions (Fig. S7h, i) shared by *M. ruthenica*, *M. archiducis-nicolaiand*, and *M. platycarpos*, show different distribution patterns of repeats, which do not support the assumption. It should be noted that the mechanisms behind the generation and loss of repeats are very complex and our data is not suitable to gain a detailed knowledge of the processes at play here. Future studies involving dense taxon sampling and/or proteins and pathways implicated in recombination, selection, and repair in plastomes (e.g. [52]) should improve our understanding of plastome disruption [48, 53].

## Conclusions

In this study, we completed 75 plastomes representing 14 species of *Medicago* and 6 species from its two closely related genera (*Melilotus* and *Trigonella*) and reported a second independent IR gain in *Melilotus dentata* for the IRLC species. We detected abundant repetitive elements and extensive genomic rearrangements in these plastomes. Notably, we found that the overall repeat content is positively correlated with the degree of genomic rearrangements. Moreover, the overall repeat contents within localized hypermutation regions around genes with accelerated substitution rates were also significantly higher than that of the remaining plastome sequences. Our findings highlight the role of repetitive sequences in affecting plastome stability in the IRLC species. The plastome data generated herein provide valuable genomic resources for further investigating the plastome evolution in legumes.

## Methods

### Plant materials

We sampled 75 individuals representing 20 species of *Medicago* and its relatives *Trigonella* and *Melilotus,* including 14 *Medicago,* 2 *Trigonella*, and 4 *Melilotus* species. Our sampling included 19 species of the three genera that are distributed in China and the model species of *Medicago*, i.e. *Medicago truncatula*. For *M. truncatula,* plastomes were assembled using whole-genome resequencing data, which were downloaded from NCBI (SRR1524305 and SRR965443, https://www.ncbi.nlm.nih.gov/). For *Medicago sativa*, seeds were germinated in the greenhouse and emergent leaves from a single plant from each accession were collected. For other species, leaves were collected from the wild (Table S15). No specific permissions were required for the relevant locations/activities. We followed the Flora of China (http://www.iplant.cn/foc/) for the nomenclature system in this study, but treated *T. arcuata* and *T. cancellata* as *M. arcuata* and *M. cancellata*, respectively, based on a recent study [54]. All voucher specimens were deposited in Lanzhou University. We selected 2–5 individuals from each species for whole genome sequencing and used the available plastome of *Trifolium subterraneum* (NC_011828.1) as an outgroup (Table S15).

### DNA sequencing, assembly, and annotation

The total genomic DNA was extracted using the modified CTAB procedure from the dried leaves [55], which were then sent to the BGI Genomics for sequencing. Paired-end libraries (2 × 150 bp) were constructed and sequenced using the Illumina Hiseq X-Ten Platform (Illumina, San Diego, CA). The raw whole genome reads (Table S1) were first quality checked by FASTQC [56], and the results showed that all the raw data were clean without adapter contamination. We, therefore, used raw genome data to de novo assemble the plastome of each individual using NOVOPlasty v.3.8.3 [57]. We used the relatively conserved *rbcL* chloroplast gene sequence as a seed to assemble the plastomes. For the four *Melilotus* species*,* we selected the *rbcL* sequence of *Melilotus albus* (GenBank accession: NC_041419.1) as seed. The *rbcL* sequence of *Trigonella foenum-graecum* voucher I.S*.* (GenBank accession: NC_042857.1) was used as seed for *Trigonella cachemiriana* and *Trigonella emodi*. For *Medicago polymorpha, M. truncatula, M. sativa, M. lupulina, M. minima*, and *M. falcata,* their seeds were provided by their own *rbcL* sequences (GenBank accession: NC_042848.1, NC_003119.8, NC_042841.1, NC_042847.1, NC_042849.1, and NC_032066.1). The *rbcL* sequence of *M. falcata* (GenBank accession: NC_032066.1) was used as seed for other *Medicago* species. We left other parameters as the default values (see NOVOPlasty README.md). We annotated the assembled plastomes using GeSeq [58] with MPI-MP chloroplast references and HMMER profile search. We further confirmed all tRNAs by tRNAscan-SE v.2.0.5 [59]. For confirmation, all annotations were compared with previously published plastomes of the three genera available in NCBI, and exon boundaries were manually corrected in Geneious v.10.2.6 [60]. In addition, we found an extra sequence inserted within *ycf1* in the four *Melilotus* species and *T. emodi*, and two extra sequences inserted within *accD* in *M. falcata* when comparing with the sequences of the two genes in other species. In order to validate whether these insertions were introns or not, we downloaded transcriptomic data of *M. falcata* (SRR1823822), *Melilotus albus* (SRR5115455), and *Trigonella foenum-graecum L.* (SRR8281660) from NCBI. For the other three *Melilotus* species and *T. emodi,* transcriptomic data are not available and the validation of *ycf1* in these species was based on the transcriptomic data of *Melilotus albus* and *Trigonella foenum-graecum*, respectively. We assembled these transcriptomes to get fasta sequences using trinityrnaseq-Trinity-v2.8.5 [61], then converted them to coding sequence (CDS) using TransDecoder-v5.5.0 (http://transdecoder.sf.net) and aligned the coding sequence (CDS) with our gene sequences (both exons and introns) via ncbi-blast-2.10.0+ [62]. In this way, we confirmed the authenticity of intron gain and corrected intron boundaries. The results showed that the intron gain of *ycf1* was true in *T. emodi* (intron boundaries were also true) but false in the four *Melilotus* species. In addition, the intron gain of *accD* in *M. falcata* was true (it does have two introns) but the intron boundaries were different from the original result. The visual images of the annotations of all species were generated by OGDRAW v.1.3.1 [63] (https://chlorobox.mpimp-golm.mpg.de/OGDraw.html). All the plastomes of the 75 individuals were newly assembled and deposited in the Genome Warehouse of CNCB-NGDC (under BioProject accession PRJCA005341).

### Inversion inference and repeat content estimate

Inversions were identified according to the arrangement of locally colinear blocks (LCB) among the newly assembled plastomes of 20 species that were estimated using progressiveMauve v.2.4.0 [64]. In progressiveMauve alignments, we selected *Wisteria floribunda,* an early diverging IRLC taxon, as the reference to identify inversions.

We calculated the repeat content of all the 75 plastomes. Tandem Repeats Finder v.4.09 [65] was used to characterize tandem repeats with the following parameters: Match of 2, Mismatch and Delta of 7, PM of 80, PI of 10, Minscore of 50, and MaxPeriod of 500. We used REPuter program [66] to identify dispersed repeats with a minimum repeat size of 30 bp and identity of no less than 90% (hamming distance equal to 3). Furthermore, we wrote a custom Perl script to eliminate the influence of nested or overlapping repeats in subsequent analysis. We only considered one IR copy for the species containing two copies of IR, both in the inversion inference and repeat content estimation.

### Comparative plastome analysis

Because structural variations among individuals within each species were almost the same (see Results), we selected 20 individuals representing all the 20 species (see the selected individuals in Table S15) for comparative analysis. To display interspecific variations graphically, full alignments with annotations of the 20 species were plotted using mVISTA [67] in the Shuffle-LAGAN mode with the annotation of *M. falcata* as a reference. To detect the sequence divergence and determine highly divergent regions of the 20 species, we used the python script “get_annotated_regions_from_gb.py” (https://github.com/Kinggerm/PersonalUtilities/) [68] to automatically extract all annotated regions and regions between annotations of the 20 plastomes, and aligned all regions using MAFFT v.7.453 [69]. We used the python script “concatenate_fasta.py” (https://github.com/Kinggerm/PersonalUtilities/) [68] to concatenate the alignments of the separate loci. Finally, we constructed three datasets, which included concatenated coding regions (PC), the concatenated noncoding loci (PN), and whole plastome (PCN). Subsequently, the nucleotide diversity (pi) of each coding gene and non-coding regions (i.e. PC, PN, and PCN) was calculated using DnaSP v.6 [70].

### Phylogenetic inference

To reconstruct the phylogenetic relationships among *Medicago* and its relatives *Trigonella* and *Melilotus,* we selected the same 20 individuals as mentioned above and used *Trifolium subterraneum* (NC_011828.1) as an outgroup. The coding sequences (CDS) of 73 protein-coding genes (PCGs) shared across the 21 species (Table S16) were extracted from each plastome using the python script “get_annotated_regions_from_gb.py” (https://github.com/Kinggerm/PersonalUtilities/) [68]. Each region was individually aligned using MAFFT v7.453 [69]. Then we used the python script “concatenate_fasta.py” (https://github.com/Kinggerm/PersonalUtilities/) [68] to concatenate the alignments. FASTA files were converted to PHYLIP format using ClustalW v.2.1 [71]. Molecular phylogenetic analysis was conducted by Maximum likelihood (ML) analysis using PhyML 3.1 [72] with 100 bootstrap replicates based on the best-fit model 012003 + I + G + F estimated by jModeltest v.2.1.7 [73].

### dN and dS analysis

Nonsynonymous (dN) and synonymous (dS) substitution rates were calculated using “yn00” from the PAML4.8 package [74] for five coding regions: *accD*, *clpP*, *ycf1*, *rbcL*, and *matK*. We then downloaded the sequences of these five genes of the IRLC species from NCBI (taxon names and accession numbers are listed in Table S17) and constructed ML trees using RAxML [75] with a general time-reversible model GTR + G + I and 100 bootstrap replicates for each of the five genes. The ML trees, which were generated using RAxML, were used as the constraint trees for five genes during the branch-specific dN and dS rate estimation in PAML4.8/codeml free-ratio model (model = 1).

## Supplementary Information

**Additional file 1 **: **Table S1.** The plastome assembly, annotation information, and distributions of genomic rearrangements for the 75 individuals. **Table S2.** Detailed information of repeat content for the 75 individuals. Repeat content for the three IR regained plastomes were calculated using only one IR copy. **Table S3.** Repeats mediated tRNA duplicates. For dispersed repeats, F: forward repeat; C: complement repeat; P: palindromic repeat; R: reverse repeat, and the numbers after the colon represent length of dispersed repeats. For tandem repeats, the numbers before the colon represent length of tandem repeats, the content after the colon represent unit size × copy number. **Table S4.** Repeats around endpoints of inversions. For dispersed repeats, F: forward repeat; C: complement repeat; P: palindromic repeat; R: reverse repeat, and the numbers after the colon represent length of dispersed repeats. For tandem repeats, the numbers before the colon represent length of tandem repeats, the content after the colon represent unit size × copy number. Palindromic repeat (P) are marked in red. **Table S5.** Palindromic repeat sequences around endpoints of inversions. **Table S6.** Repetitive DNA in the acquired introns. **Table S7.** Nucleotide diversity (pi) for different genes, intergenic regions, and datasets. PC, plastid coding regions; PN, plastid noncoding regions; PCN, the whole plastome. Eight highly divergent coding regions (π > 0.04) and 16 highly divergent non-coding regions (π > 0.1) are marked in red. **Table S8.** Sequence divergence in accD among the IRLC species. **Table S9.** Sequence divergence in clpP among the IRLC species. **Table S10.** Sequence divergence in ycf1 among the IRLC species. **Table S11.** Sequence divergence in matK among the IRLC species. **Table S12.** Sequence divergence in rbcL among the IRLC species. **Table S13.** Information of repetitive elements for the coding sequence (CDS) of three genes (accD, clpP and ycf1) with accelerated substitution rates and two relatively conserved genes (matK and rbcL). There is no coding sequence (CDS) in accD for *M. polymorpha* because it is a pseudogene (truncated sequence). **Table S14.** Percent repetitive DNA of the three localized hypermutation regions around the three genes (accD, clpP, and ycf1) with accelerated substitution rates and the remaining plastome sequences. **Table S15.** Locations of the 75 individuals representing 20 Medicago, Trigonella, and Melilotus species. The individuals for which were planted in laboratory are marked by asterisks. Plastomes of *Medicago truncatula* were assembled from whole-genome resequencing data downloaded from NCBI (SRR1524305 and SRR965443). The outgroup was downloaded from NCBI (NC_011828.1). The individuals for which were chosen as the representatives of each species are marked in red. **Table S16.** The 73 protein-coding genes (CDS) shared across 21 taxa included in the phylogenetic analysis. **Table S17.** Taxa included in the synonymous and nonsynonymous divergence analyses of accD, clpP, ycf1, matK, and rbcL. (√) adopt in analysis, (−) not available in NCBI and not adopt in analysis.

**Additional file 2 **: **Figure S1.** Plastome maps for the 20 *Medicago*, *Trigonella*, and *Melilotus* species. Genes shown outside the circle are transcribed clockwise and those inside are transcribed counter clockwise. Genes belonging to different functional groups are color-coded. The dark gray area in the inner circle indicates GC content and the thick black line shows the extent of different regions. LSC: large single copy; SSC: small single copy; IRA: inverted repeat A; IRB: inverted repeat B. The two areas enclosed by red boxes in (d) indicate small IRs in *Medicago lupulina*. In (k), red arrows No. 1 and 2 outside the circle point to the breakpoints of the inverted region involving all genes from *ycf1* to *rpl20*. Gene order in the *M. truncatula* 02 ptDNA between the arrows is in the reverse orientation and its length is ~ 44-kb (44,228 bp). Below the map are shown the alignments of 24-bp incomplete inverted repeats in the inversion endpoints in the *M. truncatula* 02 and cognate sequences in *M. truncatula* 01. **Figure S2.** IRs alignment within the four *Melilotus dentata* individuals and the repetitive elements of indel regions in *Melilotus dentata* 02 and *Melilotus dent*ata 03. Yellow bar represents protein coding gene; red bar represents rRNA gene; pink bar represents tRNA gene. Numerals above indicate nucleotide positions within the repeat alignment indicate the length of an indel within the IR. Mismatches are indicated by colored blocks and identical bases are gray. Mean pairwise identity over all pairs in each alignment column is indicated by the histogram: green 100%. IRA: inverted repeat A, IRB: inverted repeat B. Different repetitive elements were marked with different colored boxes. **Figure S3.** Mauve (Multiple Alignment of Conserved Genomic Sequence with Rearrangements) alignment of the plastomes of the 20 species using plastome of *Wisteria floribunda* as a reference. **Figure S4.** Confirmation of tRNA duplication in five species. Plastome sequences were mapped to themselves which contain all copies of the replicated tRNA (lower) and plastome sequences which contain a single copy of the replicated tRNA (upper). The scale at the left reports the depth of sequences, which is indicated graphically by the blue histogram. **Figure S5.** The degree of genomic rearrangements shows significant positive correlation with (a) dispersed repeats and (b) tandem repeats. **Figure S6.** The mechanism of tRNA duplication detected in five species. Both (a) and (b) contain a tandem repeat and two forward repeats that duplicate *trnV*-GAC twice. The three species in (c) contain a tandem repeat that duplicates the *trnN*-GUU gene. Thick black lines represent double stranded DNA. Pink boxes represent gene sequences, red and blue boxes represent tandem repeats, green boxes represent forward repeats and numbers indicate the length of repeats. **Figure S7.** Repeats around endpoints of all inversions. Red boxes represent regions of inversion, orange boxes represent protein-coding genes, and pink boxes represent tRNA genes. Black lines above double stranded DNA represent tandem repeats. Black lines below double stranded DNA represent dispersed repeats. The palindromic repeats are indicated by red lines. **Figure S8.** Repetitive DNA in the acquired introns. Yellow boxes represent exons. The lines between the yellow boxes represent acquired introns. The red content represents repetitive sequences. **Figure S9.** Comparison of the 20 plastomes using the annotation of *Medicago falcata* as a reference. The vertical scale indicates the percentage of identity, ranging from 50 to 100%. The horizontal axis indicates the coordinates within the plastomes. Genomic regions are color-coded as conserved non-coding sequences (CNS), exons, and tRNA or rRNA. **Figure S10.** Significance test for nonsynonymous (dN) (upper) and synonymous (dS) (lower) substitution rates of *clpP*, *accD*, and *ycf1* compared to *rbcL* for all the comparisons within both IRLC taxa we have found (left) and our own 20 species (right). ****, *P* < 0.0001 (T-test). Detailed information can be found in Tables S8–10 and Table S12. **Figure S11.** Synonymous and nonsynonymous divergence in the IRLC species for five chloroplast genes: *matK* (a), *rbcL* (b), *accD* (c), *clpP* (d), and *ycf1* (e). Shown are dN (left) and dS (right) trees resulting from a codon-based maximum likelihood (ML) analyses using RAxML, rooted using three Robinioid sequences (non-IRLC species): *Lotus japonicus*, *Sesbania grandiflora,* and *Robinia pseudoacacia*. The species are in the same order from top to bottom in the dN and dS trees of each gene.

## Data Availability

The following data were downloaded from NCBI (https://www.ncbi.nlm.nih.gov/): whole-genome resequencing data of *Medicago truncatula* (SRR1524305 and SRR965443); transcriptomic data of *M. falcata* (SRR1823822), *Melilotus albus* (SRR5115455) and *Trigonella foenum-graecum L.* (SRR8281660); plastome sequence of *Trifolium subterraneum* (NC_011828.1); *rbcL* chloroplast gene sequences of *Medicago polymorpha, M. truncatula, M. sativa, M. lupulina, M. minima, M. falcata, Melilotus albus* and *Trigonella foenum-graecum* (NC_042848.1, NC_003119.8, NC_042841.1, NC_042847.1, NC_042849.1, NC_032066.1, NC_041419.1, and NC_042857.1); coding sequences of *accD*, *clpP*, *ycf1*, *rbcL,* and *matK* of the IRLC species included in our study (taxon names and accession numbers are listed in Table S17). All the plastomes of the 75 individuals were newly assembled and deposited into the Genome Warehouse of CNCB-NGDC (under BioProject accession PRJCA005341). All of the parameters, program versions, and perl scripts in this study are available at https://github.com/ShuangWu888/plastomes_of_Medicago_and_its_relatives.

## Abbreviations

*M.*: *Medicago*
*T.*: *Trigonella*
IRLC: Inverted repeat lacking clade
cpDNA: Chloroplast DNA
SC: Single-copy
LSC: Large single copy
SSC: Small single copy
IR: Inverted repeat
INDELs: Insertions and deletions
CDS: Coding sequence
PCGs: Protein-coding genes
tRNA: Transfer RNA
rRNA: Ribosomal RNA
PC: Plastome coding sequence
PN: Plastome noncoding sequence
PCN: Whole plastome sequence
pi: Nucleotide diversity
dN: Nonsynonymous substitution rate
dS: Synonymous substitution rate
P: *P*-value in statistical tests
TIC: Translocon on the inner chloroplast membrane
