# Supplementary material for: Extensive genomic rearrangements mediated by repetitive sequences in plastomes of Medicago and its relatives
Source: BMC Plant Biol. 2021 Sep 14;21:421. doi: 10.1186/s12870-021-03202-3 (PMC8438982; doi:10.1186/s12870-021-03202-3)
Supplement: Supplementary file 2 — Additional file 2 : Figure S1. Plastome maps for the 20 Medicago, Trigonella, and Melilotus species. Genes shown outside the circle are transcribed clockwise and those inside are transcribed counter clockwise. Genes belonging to different functional groups are color-coded. The dark gray area in the inner circle indicates GC content and the thick black line shows the extent of different regions. LSC: large single copy; SSC: small single copy; IRA: inverted repeat A; IRB: inverted repeat B. The two areas enclosed by red boxes in (d) indicate small IRs in Medicago lupulina. In (k), red arrows No. 1 and 2 outside the circle point to the breakpoints of the inverted region involving all genes from ycf1 to rpl20. Gene order in the M. truncatula 02 ptDNA between the arrows is in the reverse orientation and its length is ~ 44-kb (44,228 bp). Below the map are shown the alignments of 24-bp incomplete inverted repeats in the inversion endpoints in the M. truncatula 02 and cognate sequences in M. truncatula 01. Figure S2. IRs alignment within the four Melilotus dentata individuals and the repetitive elements of indel regions in Melilotus dentata 02 and Melilotus dentata 03. Yellow bar represents protein coding gene; red bar represents rRNA gene; pink bar represents tRNA gene. Numerals above indicate nucleotide positions within the repeat alignment indicate the length of an indel within the IR. Mismatches are indicated by colored blocks and identical bases are gray. Mean pairwise identity over all pairs in each alignment column is indicated by the histogram: green 100%. IRA: inverted repeat A, IRB: inverted repeat B. Different repetitive elements were marked with different colored boxes. Figure S3. Mauve (Multiple Alignment of Conserved Genomic Sequence with Rearrangements) alignment of the plastomes of the 20 species using plastome of Wisteria floribunda as a reference. Figure S4. Confirmation of tRNA duplication in five species. Plastome sequences were mapped to themselves whi [file 12870_2021_3202_MOESM2_ESM.docx]

**Figure S1.** Plastome maps for the 20 *Medicago*, *Trigonella*, and *Melilotus* species. Genes shown outside the circle are transcribed clockwise and those inside are transcribed counter clockwise. Genes belonging to different functional groups are color-coded. The dark gray area in the inner circle indicates GC content and the thick black line shows the extent of different regions. LSC: large single copy; SSC: small single copy; IRA: inverted repeat A; IRB: inverted repeat B. The two areas enclosed by red boxes in (d) indicate small IRs in *Medicago lupulina*. In (k), red arrows No. 1 and 2 outside the circle point to the breakpoints of the inverted region involving all genes from *ycf1* to *rpl20*. Gene order in the *M. truncatula* 02 ptDNA between the arrows is in the reverse orientation and its length is ~44-kb (44,228bp). Below the map are shown the alignments of 24-bp incomplete inverted repeats in the inversion endpoints in the *M. truncatula* 02 and cognate sequences in *M. truncatula* 01.

**Figure S2.** IRs alignment within the four *Melilotus dentata* individuals and the repetitive elements of indel regions in *Melilotus dentata* 02 and *Melilotus dent*ata 03. Yellow bar represents protein coding gene; red bar represents rRNA gene; pink bar represents tRNA gene. Numerals above indicate nucleotide positions within the repeat alignment indicate the length of an indel within the IR. Mismatches are indicated by colored blocks and identical bases are gray. Mean pairwise identity over all pairs in each alignment column is indicated by the histogram: green 100%. IRA: inverted repeat A, IRB: inverted repeat B. Different repetitive elements were marked with different colored boxes.

**Figure S3.** Mauve (Multiple Alignment of Conserved Genomic Sequence with Rearrangements) alignment of the plastomes of the 20 species using plastome of *Wisteria floribunda* as a reference.

**Figure S4.** Confirmation of tRNA duplication in five species. Plastome sequences were mapped to themselves which contain all copies of the replicated tRNA (lower) and plastome sequences which contain a single copy of the replicated tRNA (upper). The scale at the left reports the depth of sequences, which is indicated graphically by the blue histogram.

**Figure S5.** The degree of genomic rearrangements shows significant positive correlation with (a) dispersed repeats and (b) tandem repeats.


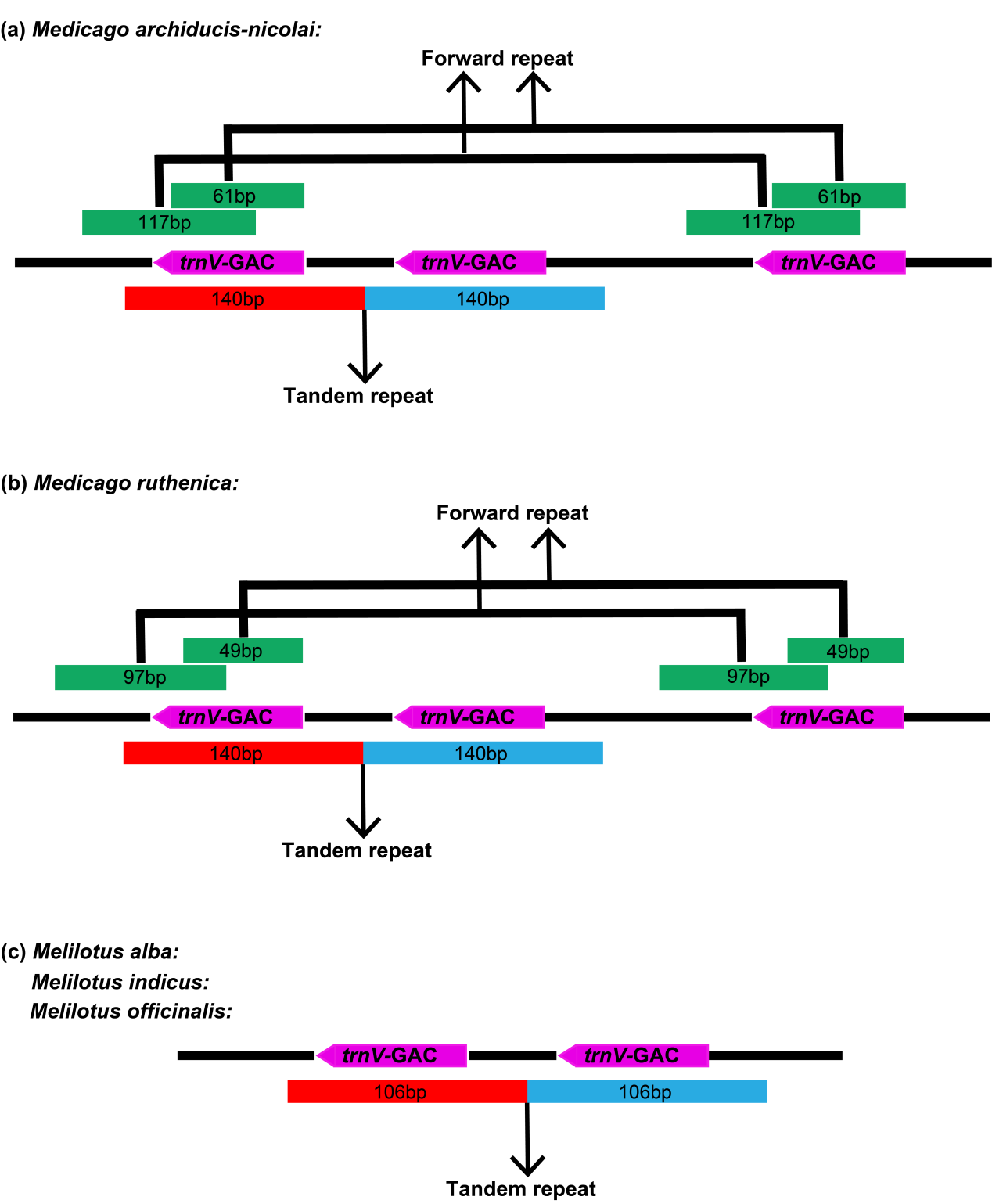


**Figure S6.** The mechanism of tRNA duplication detected in five species. Both (a) and (b) contain a tandem repeat and two forward repeats that duplicate *trnV*-GAC twice. The three species in (c) contain a tandem repeat that duplicates the *trnN*-GUU gene. Thick black lines represent double stranded DNA. Pink boxes represent gene sequences, red and blue boxes represent tandem repeats, green boxes represent forward repeats and numbers indicate the length of repeats.

**Figure S7.** Repeats around endpoints of all inversions. Red boxes represent regions of inversion, orange boxes represent protein-coding genes, and pink boxes represent tRNA genes. Black lines above double stranded DNA represent tandem repeats. Black lines below double stranded DNA represent dispersed repeats. The palindromic repeats are indicated by red lines.

**Figure S8.** Repetitive DNA in the acquired introns. Yellow boxes represent exons. The lines between the yellow boxes represent acquired introns. The red content represents repetitive sequences.

**Figure S9.** Comparison of the 20 plastomes using the annotation of *Medicago falcata* as a reference. The vertical scale indicates the percentage of identity, ranging from 50 to 100%. The horizontal axis indicates the coordinates within the plastomes. Genomic regions are color-coded as conserved non-coding sequences (CNS), exons, and tRNA or rRNA.

**Figure S10.** Significance test for nonsynonymous (dN) (upper) and synonymous (dS) (lower) substitution rates of *clpP*, *accD*, and *ycf1* compared to *rbcL* for all the comparisons within both IRLC taxa we have found (left) and our own 20 species (right). ****, *P* < 0.0001 (T-test). Detailed information can be found in Tables S8-10 and Table S12.

**Figure S11.** Synonymous and nonsynonymous divergence in the IRLC species for five chloroplast genes: *matK* (a), *rbcL* (b), *accD* (c), *clpP* (d), and *ycf1* (e). Shown are dN (left) and dS (right) trees resulting from a codon-based maximum likelihood (ML) analyses using RAxML, rooted using three Robinioid sequences (non-IRLC species): *Lotus japonicus*, *Sesbania grandiflora,* and *Robinia pseudoacacia*. The species are in the same order from top to bottom in the dN and dS trees of each gene.
